# Redox balance and immunity of piglets pre- and post-*E. coli* challenge after treatment with hemp or fish oil, and vitamin E

**DOI:** 10.1038/s41598-024-61927-1

**Published:** 2024-05-14

**Authors:** Pernille A. Madsen, Søren K. Jensen, Charlotte Lauridsen

**Affiliations:** Department of Animal and Veterinary Sciences, Aarhus University, AU Viborg-Research Centre Foulum, Blichers Allé 20, 8830 Tjele, Denmark

**Keywords:** Antioxidants, Biomarkers, Enteric infection, Fatty acid composition, Oxidative stress, Pigs, Infection, Inflammation

## Abstract

This study investigated the influence of polyunsaturated fatty acid composition and vitamin E supplementation on oxidative status and immune responses in weanling piglets pre- and post-*E. coli* challenge. Suckling piglets (n = 24) were randomly selected from two litters for an oral supplementation (1 mL/day) with fish oil or hemp oil and vitamin E supplementation (60 mg natural vitamin E/mL oil) from day 10 to 28 of age. At day 29 and 30 of age, each piglet was orally inoculated with 6.7 × 10^8^ and 3.96 × 10^8^ CFU of F4 and F18 *E. coli*, respectively. Blood was sampled from all piglets on day 28 before *E. coli* challenge and on day 35 of age to investigate immunological and oxidative stress markers in plasma. One week after weaning and exposure to *E. coli*, a general reduction in the α-tocopherol concentration and activity of GPX1 was obtained. Vitamin E supplementation lowered the extent of lipid peroxidation and improved the antioxidative status and immune responses after *E. coli* challenge. Hemp oil had the greatest effect on antioxidant enzyme activity. Provision of hemp oil and vitamin E to suckling piglets may reduce the incidence of post-weaning diarrhea.

## Introduction

Several studies have associated enteric infection in pigs with oxidative stress and inflammatory reactions^1,2^. Intestinal oxidative stress plays a crucial role in the early stage of intestinal injury by inducing intestinal barrier dysfunction thereby triggering immune imbalance and inflammation^3,4^. At weaning, the intestinal barrier function is disturbed resulting in increased exposure of the mucosa to bacteria and risk of infection with pathogenic bacteria such as *E. coli* F4 and F18 leading to oxidative stress and inflammation^5^.

Low levels of enzymatic antioxidants and vitamins have been reported in subjects with oxidative stress related diseases^6^. One strategy to minimize inflammatory mediator production is to prevent oxidative stress by enhancing the antioxidant defense mechanisms via provision of dietary antioxidative micronutrients^7,8^. Provision of antioxidants has been shown to restore redox balance and thereby reduce intestinal damage and maintaining a healthy gastrointestinal tract^1^. In addition, polyunsaturated fatty acids (PUFAs) which are prone to oxidative reactions, low antioxidative status such as reduced concentration of vitamin E and vitamin C upon weaning of pigs^9^, and/or impaired synthesis of enzymatic antioxidants (superoxide dismutase (SOD) and glutathione peroxidase-1 (GPX1)) may promote oxidative reactive reactions, and influence the immune response during infection. Furthermore, the fatty acid composition of fat sources has been reported to mitigate oxidative stress and inflammatory reactions^10,11^. Fatty acids including n-3 fatty acids have shown to inhibit the over-release of intestinal inflammatory mediators especially pro-inflammatory cytokines^12,13^. Patterson et al.^14^ showed that *E. coli*-challenged piglets weaned from sows provided 2% conjugated linoleic acid (C18:2n-6) had reduced intestinal inflammation and increased immunoglobulin G (IgG) and immunoglobulin A (IgA) compared to piglets weaned from control sows. Fish oil is well-known for the content of n-3 longer chained PUFAs (LCPUFAs) including eicosapentaenoic acid (EPA) and docosahexaenoic acid (DHA), and incorporation of these fatty acids into piglets’ alveolar macrophages reduced inflammatory responses when compared to sunflower oil having a high content of linoleic acid^15^. Thus, supplementation of n-3 fatty acids as well as conjugated linoleic acid (C18:2) has been suggested as a practical strategy to enhance overall gut health of piglets due to anti-inflammatory mechanisms. However, since n-3 LCPUFAs are highly prone to oxidation, other fat sources having high content of linolenic derived metabolites may enhance the capability of synthesis of EPA and DHA. Hemp (*Cannabis sativa* L.) is an old culture plant that is known for its high content of bioactive compounds with antioxidant properties. Furthermore, hempseed oil contains a unique fatty acid profile compared to other vegetable oils, which besides linoleic acid (18:2n-6) and α-linolenic acid (ALA; 18:3n-3) also contains γ-linolenic acid (GLA; C18:3n-6) and stearidonic acid (SDA; C18:4n-3)^16^. Several phytomolecules including tocopherols, phenols, polyphenols and lignanamides have been identified in hempseed oil and have been demonstrated to be important for cell protection against oxidative stress^17,18^.

The aim of the current study was to investigate the influence of oil sources with different proportion of n-6 and n-3 PUFAs, and supplementation of an antioxidant in the form of natural vitamin E on oxidative stress and immune responses during enteric infection. The treatments in the present study include fish oil, which is rich in n-3 PUFAs, and hemp oil (*Cannabis sativa* L.), which has a high content of both n-6 and n-3 PUFAs^19,20^ and contains bioactive compounds with antioxidant properties^21^. It was hypothesized that the increased proportion of LCPUFAs and low antioxidant concentration would enhance the susceptibility to oxidative stress and impair immune responses. The oil treatments were provided to suckling piglets, i.e., prior to weaning to influence the fatty acid composition and vitamin E concentration of immune cells at weaning. All piglets were exposed to an *E. coli* challenge to mimic common challenges in practice post-weaning including induction of inflammation and oxidative stress^5^.

## Materials and methods

### Animal experimental conditions

The animal experiment was performed with permission from The Danish Animal Experimentation Inspectorate, and the experiment was conducted according to a license obtained from the Danish Animal Experiments Inspectorate, Danish Veterinary and Food Administration, Ministry of Food, Agriculture and Fisheries (# 2017-15-0201-01270). In addition, the study was performed in accordance with the ARRIVE guidelines (Animal Research: Reporting of In Vivo Experiments). The pigs used in the study were monitored by proper veterinary surveillance throughout the experiment. Housing and rearing of the animals followed Danish laws and regulations for humane care and use of animals in research, i.e., the health of the pigs was monitored daily, and illness was treated by trained personnel.

### Experimental design, recordings, and sample collection

The experiment included suckling piglets (n = 24) bred from two genetically homozygous *E. coli* F4 and F18 susceptible sows (Danish Landrace × Yorkshire) mated with a Duroc boar from the herd at AU Viborg Research Centre, Foulum, Aarhus University. At day 10 of age, the piglets were ear-labelled and allocated to treatments. Two to three piglets from each of the two litters were randomly selected to one of four oil treatments (n = 6 piglets per treatment). The treatments consisted of an oral supplementation (1 mL/day) with fish oil or hemp oil, and vitamin E supplementation (60 mg natural vitamin E/mL oil as a combination of α-, γ- and δ-tocopherol). The treatments were as follows: fish oil (Fish); fish oil supplemented with vitamin E (Fish + vitamin E); hemp oil (Hemp); hemp oil supplemented with vitamin E (Hemp + vitamin E). Hemp oil (Nørding Olier I/S, Silkeborg, Denmark) and fish oil (ESKIO-3 Pure^®^, Denmark) were commercially available. The ESKIO-3 Pure^®^ was enriched with a tocopherol mixture (amount was not declared). Provision of the treatments to the piglets was carried out orally via a syringe from day 10 to day 28 of age as a supplement to sow milk. At weaning (at day 28 of age), the piglets were provided a standard weaner diet without zinc oxide mixed at AU Viborg—Research Centre Foulum. Sows were provided a standard sow lactation diet supplemented with 192 mg dl-α-tocopheryl acetate per kg feed (Die Plus U, DLG, Denmark).

Piglets were individually weighed at birth and weekly until weaning and were weighed again on day 35 of age. The health status of the animals was recorded daily, and dead piglets were noted as well as any medical treatment. At weaning on day 28 of age, two piglets from each treatment (n = 8) were euthanized and samples were collected from ileum including tissue and mucosal scrapings. Euthanatizing of the pigs was carried out using a non-penetrating captive bolt gun. Of the remaining piglets (n = 16), four piglets from each treatment were selected and housed pairwise in a pen where they were all offered the same standard weaner diet. On day 29 and 30 of age, all remaining piglets were orally inoculated with an equal mix of *E. coli* F4 0149 and *E. coli* F18 038 strains, which consisted of (by bacterial counting of the inoculum) 6.7 × 10^8^ and 3.96 × 10^8^ CFU of F4 and F18, respectively. Feces samples were collected daily after weaning and fecal score was registered using a scale from 1–7 (fecal score 4–7 indicates diarrhea) according to Carstensen et al.^22^. The piglets were euthanized 5 days after *E. coli* inoculation (on day 35 of age) and tissue samples were obtained from the ileum. All intestinal tissue samples were stored at − 80 °C until analysis. Blood was sampled from all piglets at day 7, 14, 21 and 28 of age (before weaning from the dam and *E. coli* challenge), and at the end of the experiment on day 35 of age. All blood samples were collected in sodium and heparinized Vacutainers (Vacuette, Greiner Bio-One GmbH, Kremsmünster, Austria). Plasma was extracted by centrifugation at 2000*g* and subsequently stored at − 80 °C until analysis of fatty acid composition, α-tocopherol, immunological parameters including cytokines and immunoglobulin A, G and M, as well as oxidative stress markers.

### Laboratory analyses

Concentration of fatty acids and vitamin E was analyzed in plasma and ileal tissue. Immunological responses were examined by analysis of immunoglobulins and cytokines in plasma samples after in vitro LPS stimulation. In addition, oxidative stress responses were examined by analysis of oxidative stress markers in plasma samples. Below, laboratory methods are described in detail for the analyses performed.

### Lipid and fatty acid analyses

Lipid content and fatty acid composition were analyzed by a modified Bligh and Dyer extraction procedure using water–methanol-chloroform and acid treatment as previously described by Jensen^23^. In brief, 50 mg oil or 0.500 mL plasma were extracted with 1.5 mL (oil) 1.0 mL (plasma) of distilled water, 3.0 mL of chloroform, 3.0 mL of methanol, and 5.0 mg of C17:0 (heptadecanoic acid, Sigma-Aldrich, St. Louis, MO) as the internal standard. The extracts were centrifuged for 10 min at 1558×*g*. Precisely 1.0 mL of chloroform phase was transferred to a new tube, evaporated under a nitrogen stream, and then methylated with 0.8 mL of NaOH (2%) in methanol according to Petersen and Jensen^24^. The tubes were filled with argon and transferred to an oven for 20 min at 100 °C. After cooling, 1.0 mL of boron trifluoride reagent was added, filled with argon, and placed in an oven for 45 min at 100 °C. Finally, fatty acid methyl esters were extracted with 2.0 mL of heptane and 4 mL of a saturated NaCl solution, followed by centrifugation for 10 min at 1558×*g*. A gas chromatograph (Hewlett Packard 6890, Agilent Technologies, Palo Alto, CA, USA) was used for quantifying the fatty acids as fatty acid methyl esters. The chromatograph was equipped with an auto-column injector (HP 7673), a capillary column of 60 m × 0.32 mm inner diameter and a film thickness of 0.25 µm (Omegawax 320; Supelco 4-293-415, Sigma-Aldrich), and a flame ionization detector. The initial temperature was set to 86 °C and increased to 200 °C at a rate of 2 °C per min. The temperature was then maintained at 200 °C for 5 min before increasing to the final target of 220 °C. Each peak was identified through a comparison of retention time with the external standard (GLC 68C, Nu-Prep- Check, Elysian, MN, USA).

### Analysis of α-tocopherols

Quantitative determination of α-tocopherols including α-, γ- and δ-tocopherol was carried out by HPLC after saponification and extraction into heptane as previously described by Jensen et al.^25^. In brief, plasma 0.500 mL was diluted with 2.0 mL of ethanol (96% v/v), 0.5 mL of methanol (100%), 1.0 of mL ascorbic acid (20% w/v), 0.3 mL of KOH-water (1:1, w/v) and 1.2 mL of water. Samples were saponified at 80 °C for 20 min and cooled in the dark. Tocopherol was extracted into two volumes of 5 mL of heptane, and 0.100 mL of the combined heptane phase was injected into the HPLC. All solvents used were of HPLC quality. The HPLC column for determination of tocopherols consisted of a 4.0 × 125 mm Perkin-Elmer HS-5-Silica column (Perkin-Elmer GmbH, D-7770 Überlingen, Germany). The mobile phase consisted of heptane containing 2-propanol (3.0 mL/L) and degassed with helium. The flow rate was 3.0 mL/min. A comparison of retention time and peak areas with Merck (D-6100 Damstadt, Germany) external standards were used to obtain the identification and quantification of the tocopherol. Fluorescence detection was performed with an excitation wavelength of 290 nm and an emission wavelength of 327 nm.

### *E. coli* quantification in feces

Quantification of *E. coli* in feces samples was determined by the plate count method (Columbia blood agar with sheep blood medium, Thermo Fisher Scientific, Waltham, Massachusetts, USA) followed by aerobically incubation at 37 °C overnight. Using a manual colony counter, hemolytic bacteria were counted and expressed as CFU/g feces. The detection limit was 10^4^ CFU/g feces. Serotyping of F4 and F18 *E. coli* was accomplished using the slide agglutination test with type specific antisera (SSI Diagnostica A/S, Hillerød, Denmark) on five colonies per plate. The dry matter content of feces was determined by freeze drying the samples to a constant weight.

### Immunological parameters

Blood samples were stimulated with LPS (*Escherichia* *coli* O111:B4, Sigma-Aldrich, Denmark) as described by Carstensen et al.^26^. Granulocyte-macrophage colony-stimulating factor (GM-CSF) as well as the following cytokines interferon-γ (INF-γ),

tumor necrosis factor-α (TNF-α), and interleukins (IL) IL-1α, IL-β, IL-1RA, IL-2, IL-4, IL-6, IL-8, IL-10, IL-12, and IL-18 were analyzed in plasma samples using the Milliplex Map porcine kit (Merck, Germany) on a Bio-Plex^®^ MAGPIX™ Multiplex Reader (Bio-Rad, Richmond, CA, USA). Plasma samples and mucosal scraping samples from ileum were analyzed for concentration of immunoglobulins using commercially available sandwich ELISA kit (Abnova, Bio-Rad, Richmond, CA, USA) according to manufacturer’s instructions, and the concentration of mucosal IgA was expressed as the relative amount of IgA to total protein. Total protein content in mucosa was analyzed on an autoanalyser, OpeRA™, Chemistry System (Bayer Corporation) according to the biuret method as standardised by the Technicon RA^®^ Systems^27^.

### Oxidative stress markers

Malondialdehyde (MDA) was analyzed in plasma samples as an indicator of lipid peroxidation using a competitive ELISA kit (Nordic BioSite, Sweden) via spectrophotometry/fluorimetric assay according to manufacturer’s instructions. In addition, antioxidant enzyme activity in plasma samples was investigated by analysis of GPX1 using a sandwich ELISA kit (Nordic BioSite, Sweden) via spectrophotometry/fluorimetric assay, and the enzyme activity of superoxide dismutase (SOD) was analyzed using a colorimetric activity kit (Arbor Assays, MI, USA) according to manufacturer’s instructions.

### Statistical analysis

The impact of treatments (Fish, Hemp, Fish + vitamin E, Hemp + vitamin E) and age on plasma concentrations of oxidative stress markers was examined by a linear mixed effects model using the *lme* function from the *nlme* package in R version 4.2.1^28^ according to the following equations:

1 $${Y}_{ijkl}=\mu +{\alpha }_{i}+{\beta }_{j}+{\alpha \beta }_{ij}+{\omega }_{k}+{\varepsilon }_{ijkl}$$

where ${Y}_{ijkl}$ is the dependent variable, $\mu$ is the overall mean, ${\alpha }_{i}$ is the effect of oil source ($i$ = Fish or Hemp), ${\beta }_{j}$ is the effect of vitamin E supplementation ($j$ = with or without vitamin E supplementation), ${\alpha \beta }_{ij}$ is the interaction between oil source and vitamin E supplementation, ${\omega }_{k}\sim N(0,{\sigma }_{\omega }^{2})$ is the random effect of litter $k$, ${\varepsilon }_{ijkl}\sim N(0,{\sigma }^{2})$ is the residual error and $l=1,2,\ldots ,{n}_{ik}$ is piglets within litter $k$ receiving treatment $i$.

2 $${Y}_{mokl}=\mu +{\gamma }_{m}+ {\updelta }_{o}+{\gamma \delta }_{mo}+{\omega }_{k}+{\varepsilon }_{mokl}$$

where ${Y}_{mokl}$ is the dependent variable, $\mu$ is the overall mean, ${\gamma }_{m}$ is the effect of treatment ($m$ = Fish, Hemp, Fish + vitamin E, Hemp + vitamin E), ${\updelta }_{o}$ is the effect of age ($o$ = day 28 or 35 of age), ${\gamma \delta }_{mo}$ is the interaction between vitamin E supplementation and age, ${\omega }_{k}\sim N(0,{\sigma }_{\omega }^{2})$ is the random effect of litter $k$, ${\varepsilon }_{mokl}\sim N(0,{\sigma }^{2})$ is the residual error and $l=1,2,\dots ,{n}_{mk}$ is piglets within litter $k$ receiving treatment $m$. In addition, the statistical analysis of the plasma and ileal concentration of immunological parameters and fatty acids were accomplished using the MIXED procedure in SAS (SAS Inst., Inc., Cary, NC) using Eqs. (1) and (2), respectively.

For all models (Eqs. 1 and 2), estimated least squares means (LS means) ± SEM were obtained using the emmeans package. Exploratory comparisons were carried out with emmeans on log-scale. Differences were considered significant when *P* < 0.05 and trends when *P* < 0.10.

### Ethics approval

Housing and care of the experimental animals was performed in accordance with Danish laws and regulations for humane care and use of animals in research [The Danish Ministry of Justice, Animal Testing Act (Consolidation Act number 726 of September 9, 1993, as amended by Act number 1081 on December 20, 1995)]. The Danish Animal Experimentation Inspectorate approved the study protocol and supervised the experiment (# 2017-15-0201-01270).

## Results

### Body weight

The body weight of the piglets was not affected by oil source (*P* = 0.12) or supplementation of vitamin E (*P* = 0.14) but increased with age of the piglets (*P* < 0.001); 1.35 kg ± 0.43 at birth vs. 7.84 kg ± 0.43 on day 28 of age when averaged across treatments.

### Vitamin E and fatty acid composition in oil treatments and piglets’ plasma

The fish oil contained a much higher concentration of vitamin E than hemp oil in the forms of α-tocopherol and δ-tocopherol, whereas the concentration of γ-tocopherol was similar (Table 1). In addition, for both fish oil and hemp oil, the concentration of α-tocopherol and γ-tocopherol was greater in the oil treatments when supplemented with natural vitamin E mixture, whereas the concentration of δ-tocopherol was similar.

**Table 1:** Concentration (µg/mL) of tocopherol isomer forms in the oil treatments.

Tocopherol isomer form	Fish	Fish + vitamin E	Hemp	Hemp + vitamin E
α-Tocopherol	598 ± 6.36	625 ± 10.6	25 ± 0.01	56 ± 0.71
γ-Tocopherol	472 ± 9.19	475 ± 16.9	493 ± 4.24	508 ± 2.12
δ-Tocopherol	256 ± 1.41	257 ± 0.71	9.73 ± 0.78	9.57 ± 0.85

Values are presented as mean ± SD (one replicated sample).

**Figure 1:**
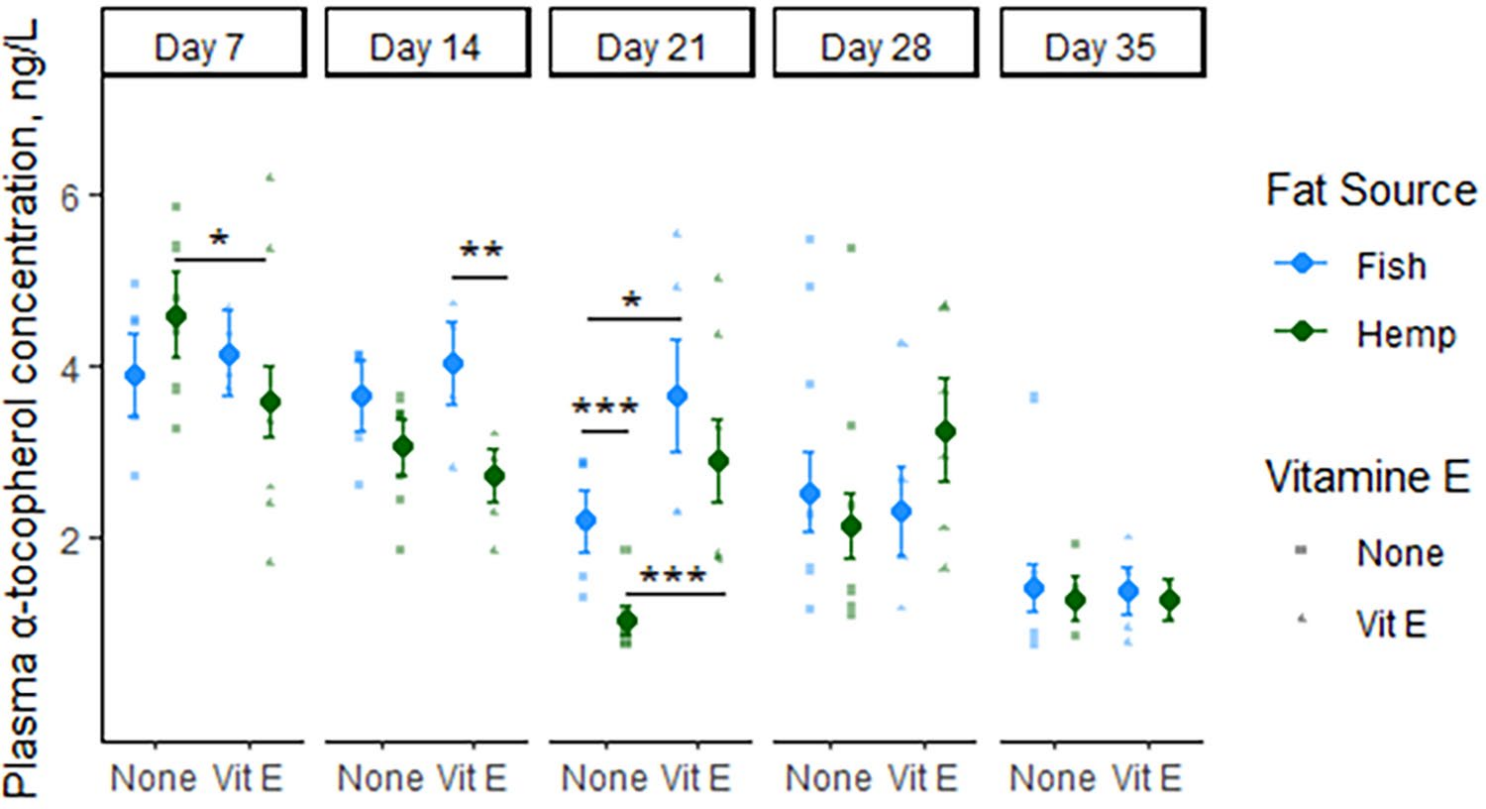
Plasma concentration of α-tocopherol on day 7, 14, 21, 28 and 35 of age in piglets provided fish oil or hemp oil supplemented with vitamin E or not. Piglets were provided the oil treatments from day 10 of age until day 28 of age. The dots represent estimated marginal means, and bars are standard errors. Statistical significance between treatments is indicated by *< 0.05, **< 0.01 and ***< 0.001.

The plasma concentration of α-tocopherol was affected by oil source (*P* < 0.05; Fig. 1) as piglets provided fish oil had higher plasma levels of α-tocopherol than piglets provided hemp oil at day 14 and 21 of age. In addition, the plasma concentration of α-tocopherol was greater in piglets receiving oil treatment and vitamin E compared to piglets provided only the oil treatment. Irrespective of treatment, all piglets had a reduction in plasma α-tocopherol from day 28 of age to day 35 of age (*P* < 0.01; 2.95 vs. 1.48 ng/L, Fig. 1). On day 7 of age, the plasma concentration of α-tocopherol was higher (*P* = 0.05) in piglets provided hemp oil without addition of vitamin E compared to piglets provided hemp oil supplemented with vitamin E, although the oil treatment had not been initiated yet (Fig. 1). No γ-tocopherol or δ-tocopherol was detected in the plasma samples of the pigs.

Hemp oil was rich in C18:2n-6 and C18:3n-3 but did not contain LCPUFAs including C20:5n-3, C22:5n-3, and C22:6n-3, whereas fish oil contained C20:5n-3, C22:6n-3, C22:5n-3, C18:2n-6, and C18:3n-3 fatty acids (Table 2). Moreover, the unsaturated/saturated and the n-6/n-3 ratio was greater in hemp oil than in fish oil, while fish oil contained a higher proportion of monounsaturated fatty acids than hemp oil.

**Table 2:** Concentration of fatty acids (%) in fish oil and hemp oil.

Fatty acid	Hemp oil	Fish oil
Saturated		
C14:0	0.04 ± 0.01	8.15 ± 0.02
C15:0	0.12 ± 0.01	0.66 ± 0.01
C16:0	5.98 ± 0.02	18.1 ± 0.01
C18:0	3.46 ± 0.02	3.63 ± 0.03
C20:0	0.97 ± 0.01	0.22 ± 0.01
C22:0	0.37 ± 0.01	0.13 ± 0.01
Total	10.9	30.9
Monounsaturated		
C16:1n-7	0.10 ± 0.01	10.1 ± 0.01
C16:1n-9	0.03 ± 0.01	0.22 ± 0.01
C18:1-n7	0.77 ± 0.01	3.21 ± 0.01
C18:1-n9	14.7 ± 0.02	9.75 ± 0.01
C20:1-n9	0.42 ± 0.01	1.02 ± 0.01
C22:1n-11	–	0.59 ± 0.01
Total	16.0	24.9
n-6 Polyunsaturated		
C18:2n-6	53.8 ± 0.04	1.50 ± 0.01
C18:3n-6	2.15 ± 0.01	0.32 ± 0.01
C20:2n-6	0.05 ± 0.01	0.16 ± 0.01
C20:3n-6	–	0.19 ± 0.01
C20:4n-6	0.01 ± 0.01	1.13 ± 0.01
C22:5n-6	–	0.15 ± 0.01
Total	56.0	3.45
n-3 Polyunsaturated		
C18:3n-3	16.3 ± 0.01	0.80 ± 0.01
C18:4n-3	0.73 ± 0.01	3.43 ± 0.01
C20:3n-3	–	0.10 ± 0.01
C20:5n-3	–	20.2 ± 0.01
C22:5n-3	–	2.20 ± 0.01
C22:6n-3	–	12.7 ± 0.01
Total	17.0	39.4
Unsaturated/saturated	8.17	2.19
n-6:n-3 ratio	3.3	0.09

Values are presented as mean ± SD (one replicated sample). Decimals are roundup to two digits.

The proportion of fatty acids was affected by oil treatment and by age of the piglets (Table 3). In general, the plasma concentration of monounsaturated fatty acids was greatest in piglets provided fish oil (*P* ≤ 0.05), while the proportion of polyunsaturated fatty acids was greatest in plasma of piglets provided hemp oil (*P* ≤ 0.07). Although hemp oil did not contain any n-3 LCPUFAs (20:3, C20:5, C22:5, C22:6), these fatty acids were present in plasma of the piglets. In addition, the plasma concentration of the analyzed fatty acids was in general increased in all piglets from day 28 of age to day 35 of age (*P* ≤ 0.04). Furthermore, no effect of treatment was observed on mucosal fatty acid concentrations or intestinal IgA concentrations after *E. coli* challenge (data not shown).

**Table 3:** Proportion (%) of fatty acids in plasma of piglets at day 28 and day 35 of age.

Day of age	28		35			*P* value	
Fatty acid	Fish	Hemp	Fish	Hemp	SEM	Oil source	Day
Saturated							
C14:0	1.31	1.40	0.55	0.53	0.17	0.66	0.01
C15:0	0.27	0.24	0.46	0.50	0.79	0.82	0.01
C16:0	25.4	26.4	18.6	17.6	1.95	0.44	0.01
C18:0	9.38	9.36	10.7	10.8	0.86	0.96	0.01
C20:0	0.09	0.07	0.13	0.16	0.03	0.83	0.01
Total	36.1	37.5	30.4	29.6	0.67	0.34	0.01
Monounsaturated							
C16:1n-7	6.43	6.83	2.43	1.48	1.10	0.79	0.01
C16:1n-9	0.46	0.41	0.62	0.52	0.09	0.01	0.01
C18:1n-7	2.49	2.26	3.01	2.47	0.41	0.01	0.01
C18:1n-9	18.0	17.5	20.7	18.2	2.49	0.05	0.03
C20:1n-9	0.11	0.11	0.20	0.23	0.04	0.83	0.01
Total	27.6	26.6	26.2	22.8	1.67	0.06	0.02
Polyunsaturated							
C18:2n-6	22.6	23.7	26.0	31.1	4.56	0.03	0.01
C18:3n-3	0.79	0.90	1.39	1.85	0.35	0.07	0.01
C18:3n-6	0.39	0.35	0.41	0.39	0.09	0.28	0.29
C20:2n-6	0.17	0.17	0.22	0.21	0.03	0.23	0.01
C20:3n-6	0.34	0.31	0.32	0.32	0.09	0.67	0.74
C20:4n-6	6.76	6.39	7.76	7.36	1.35	0.30	0.03
C20:5n-3	0.64	0.31	0.71	0.86	0.16	0.01	0.01
C22:5n-6	0.33	0.35	0.30	0.34	0.09	0.30	0.65
C22:5n-3	1.08	0.85	1.25	1.13	0.32	0.08	0.04
C22:6n-3	2.08	1.18	2.97	2.77	0.55	0.01	0.01
Total	35.3	34.6	38.6	45.9	1.75	0.29	0.01
Unsaturated/ saturated	1.75	1.65	2.25	2.34	0.06	0.47	0.01
n6:n3 ratio	6.66	10.0	5.66	6.03	0.55	0.01	0.01

Values are presented as estimated marginal means and pooled standard errors (SEM), n = 6 at day 28 of age, and n = 4 at day 35 of age. Piglets were provided oil treatments from day 10 of age to day 28 of age.

### Immunological parameters

Plasma cytokine responses obtained at the day of weaning and after in vitro* E. coli* LPS stimulation were influenced by treatments (Table 4); vitamin E supplementation increased concentration of INF-γ (*P* = 0.05) and tended to increase concentration of IL-18 (*P* = 0.07), while fish oil reduced the concentration of IL-12 (*P* = 0.05) compared to hemp oil.

**Table 4:** Concentration of cytokines (ng/mL) in plasma of piglets at day 28 of age after in vitro* E. coli* LPS stimulation.

Cytokines	Treatment				SEM	*P* value	
	Fish	Hemp	Fish + vitamin E	Hemp + vitamin E		Oil source	Vit E
GM-CSF	0.10	0.09	0.10	0.10	0.13	0.93	0.38
INF-γ	4.63	4.14	5.26	5.88	1.37	0.23	0.05
IL-1α	0.23	0.17	0.17	0.21	0.06	0.54	0.45
IL-β	7.10	6.26	6.17	6.58	1.93	0.42	0.36
IL-1RA	7.94	4.59	5.31	3.87	4.95	0.40	0.47
IL-2	0.09	0.09	0.10	0.09	0.02	0.21	0.16
IL-4	0.17	0.12	0.21	0.13	0.05	0.11	0.10
IL-6	0.94	0.78	0.70	0.94	0.30	0.45	0.52
IL-8	1.29	1.18	2.17	1.58	0.77	0.52	0.74
IL-10	0.22	0.21	0.22	0.22	0.05	0.50	0.50
IL-12	0.56	0.69	0.66	0.92	0.21	0.05	0.10
IL-18	0.66	0.63	0.99	0.79	0.24	0.24	0.07
TNF-α	0.76	0.68	0.90	1.18	0.56	0.41	0.41

Values are presented as estimated marginal means and pooled standard errors (SEM), n = 6. Piglets were provided oil treatments from day 10 of age to day 28 of age.

At day 7 of age, the plasma concentration of IgA was higher (*P* = 0.05) in piglets provided fish oil and vitamin E than in piglets provided only fish oil (Table 5), although it should be noted that the oil treatments were not provided before day 10 of age. At day 7 and 14 of age, the plasma concentration of IgG was lower in piglets provided fish oil and vitamin E than in piglets provided only fish oil (*P* = 0.01 and *P* = 0.01, respectively). In addition, at day 7 of age, piglets provided hemp oil and vitamin E had greater IgG plasma concentration than piglets provided fish oil and vitamin E (*P* = 0.02). Furthermore, at day 14, 28 and 35 of age, the plasma concentration of IgM was higher in piglets provided fish oil and vitamin E compared to piglets provided only fish oil (*P* = 0.03, *P* = 0.04 and *P* = 0.03, respectively).

**Table 5:** Effect of oil treatments on plasma concentration of immunoglobulins.

Cytokines	Treatment				SEM	*P* value	
	Fish	Hemp	Fish + vitamin E	Hemp + vitamin E		Oil source	Vit E
IgA, µg/mL							
Day 7	810	998	1473	1096	81.3	0.34	0.05
Day 14	213	242	152	251	17.8	0.41	0.58
Day 21	175	213	153	247	17.5	0.53	0.86
Day 28	157	207	210	292	19.4	0.68	0.71
Day 35	282	305	353	439	34.7	0.83	0.82
IgG, mg/mL							
Day 7	15.7	15.8	10.8	14.6	1.88	0.02	0.01
Day 14	9.81	9.39	6.73	7.88	1.15	0.29	0.01
Day 21	7.87	7.67	6.26	6.68	0.98	0.66	0.12
Day 28	4.32	5.22	4.40	4.54	0.71	0.83	0.89
Day 35	5.76	5.43	6.24	5.68	0.93	0.60	0.65
IgM, µg/mL							
Day 7	574	519	557	530	10.3	0.54	0.86
Day 14	298	387	313	232	6.66	0.22	0.03
Day 21	439	458	609	464	12.0	0.88	0.27
Day 28	515	678	817	649	13.7	0.19	0.04
Day 35	837	912	1245	800	18.1	0.03	0.03

Values are presented as estimated marginal means and pooled standard errors (SEM), n = 6 piglets on day 7, 14, 21 and 28, and n = 4 piglets on day 35 of age. Piglets were provided oil treatments from day 10 of age until day 28 of age.

### Fecal shedding of *E. coli* and fecal score

**Figure 2:**
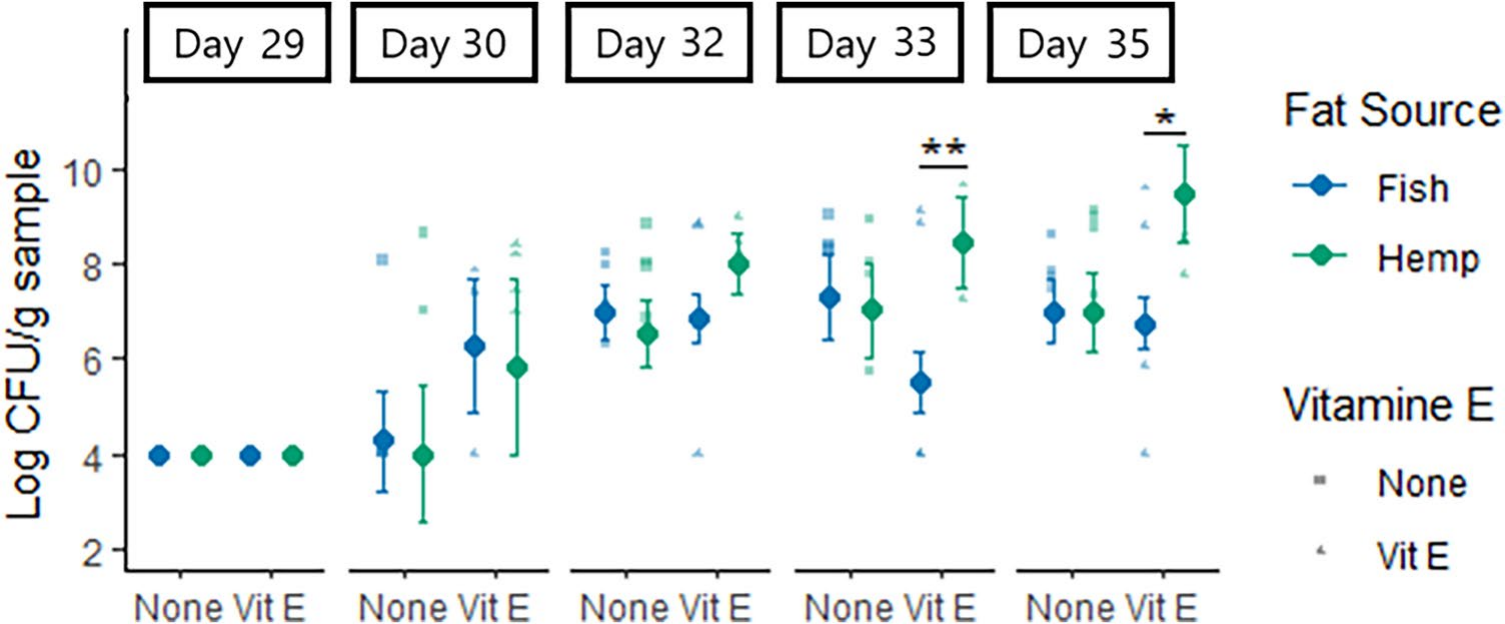
Shedding of *E. coli* (log CFU/g sample) on day 29, 30, 32, 33 and 35 of age in piglets provided fish oil or hemp oil supplemented with vitamin E or not during the suckling period (day 11 to day 28 of age). Piglets were provided the oil treatments from day 10 of age until day 28 of age. The dots represent estimated marginal means, and bars are standard errors. Statistical significance between treatments is indicated by *< 0.05 and **< 0.01. The detection limit was 10^4^ CFU/g feces.

Before *E. coli* inoculation (at day 29 of age, Fig. 2), there was no detectable F4 and F18 *E. coli* in feces of the pigs and no difference between treatments. The fecal shedding of *E. coli* was in general not affected by the oil treatments at day 30 and 32 of age. However, at day 33 and 35 of age, the fecal count was lower (*P* < 0.01 and *P* = 0.01, respectively) in pigs provided fish oil compared to pigs provided hemp oil when supplemented with vitamin E.

**Figure 3:**
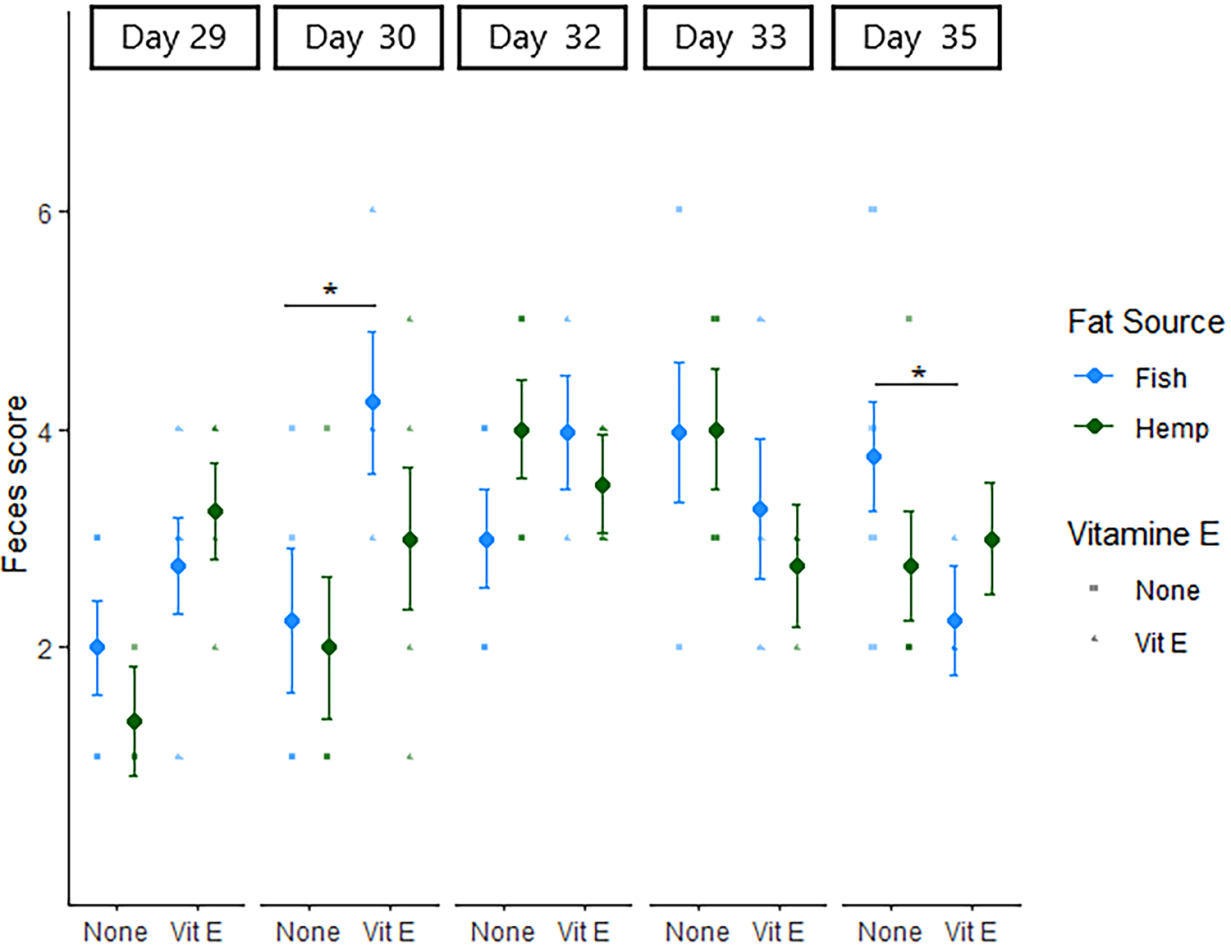
Fecal score (scale from 1–7, fecal score 4–7 indicates diarrhea) on day 29, 30, 32, 33 and 35 of age in piglets provided fish oil or hemp oil supplemented with vitamin E or not during the suckling period (day 11 to day 28 of age). Piglets were provided the oil treatments from day 10 of age until day 28 of age. The dots represent estimated marginal means, and bars are standard errors. Statistical significance between treatments is indicated by *< 0.05.

The fecal score was in general not affected by treatment (Fig. 3). However, at day 30 of age, the fecal score was higher in pigs provided fish oil supplemented with vitamin E compared to pigs provided only fish oil (*P* = 0.03), whereas at day 35 of age, the fecal score was lower in pigs provided fish oil and vitamin E compared to pigs provided only fish oil (*P* = 0.04).

### Oxidative status

**Figure 4:**
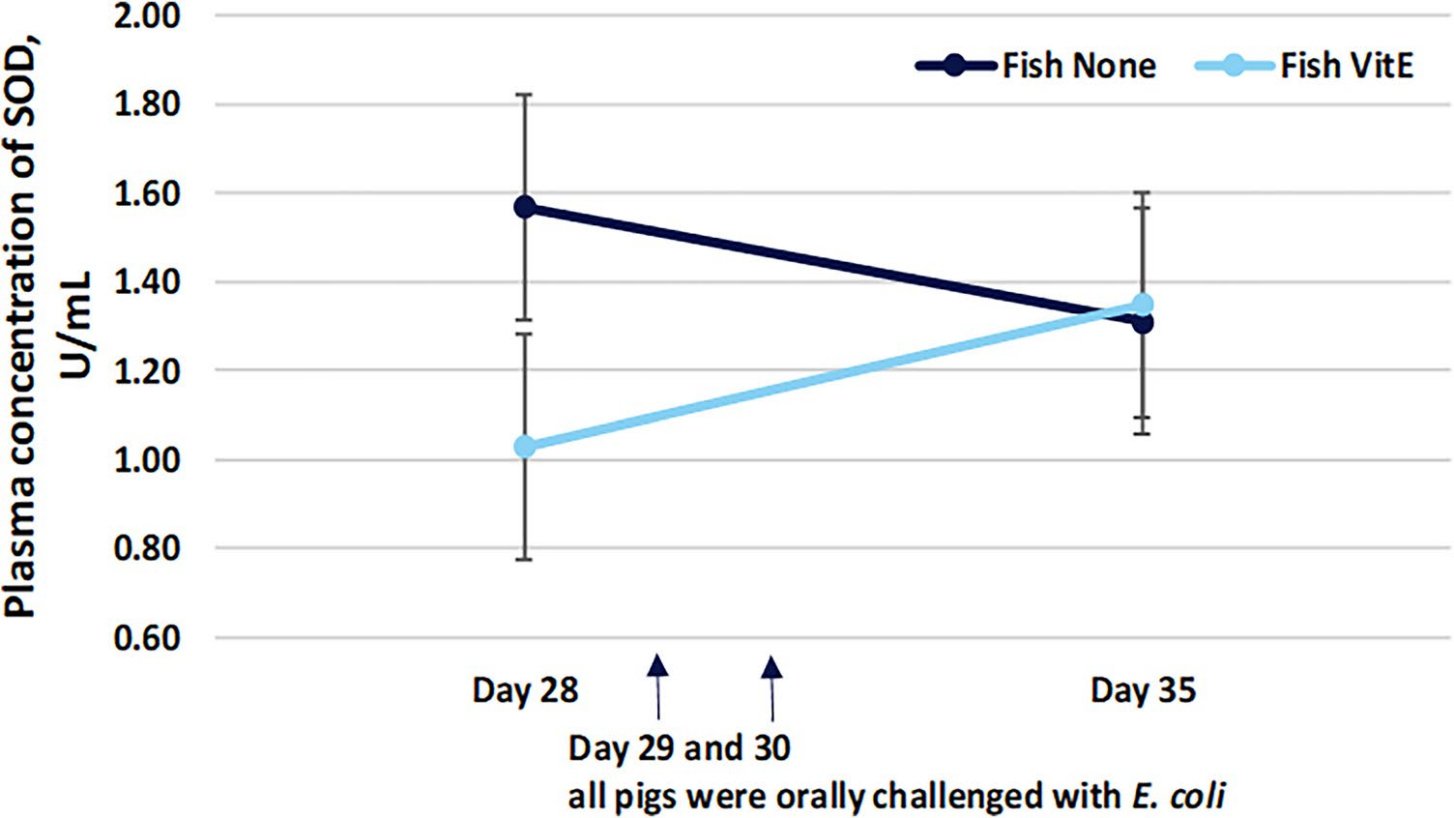
Interaction plot between day and supplementation of vitamin E showing that by combining fish oil and vitamin E, the plasma concentration of SOD increased after *E. coli* challenge. Piglets were provided the oil treatments from day 10 of age until day 28 of age.

**Figure 5:**
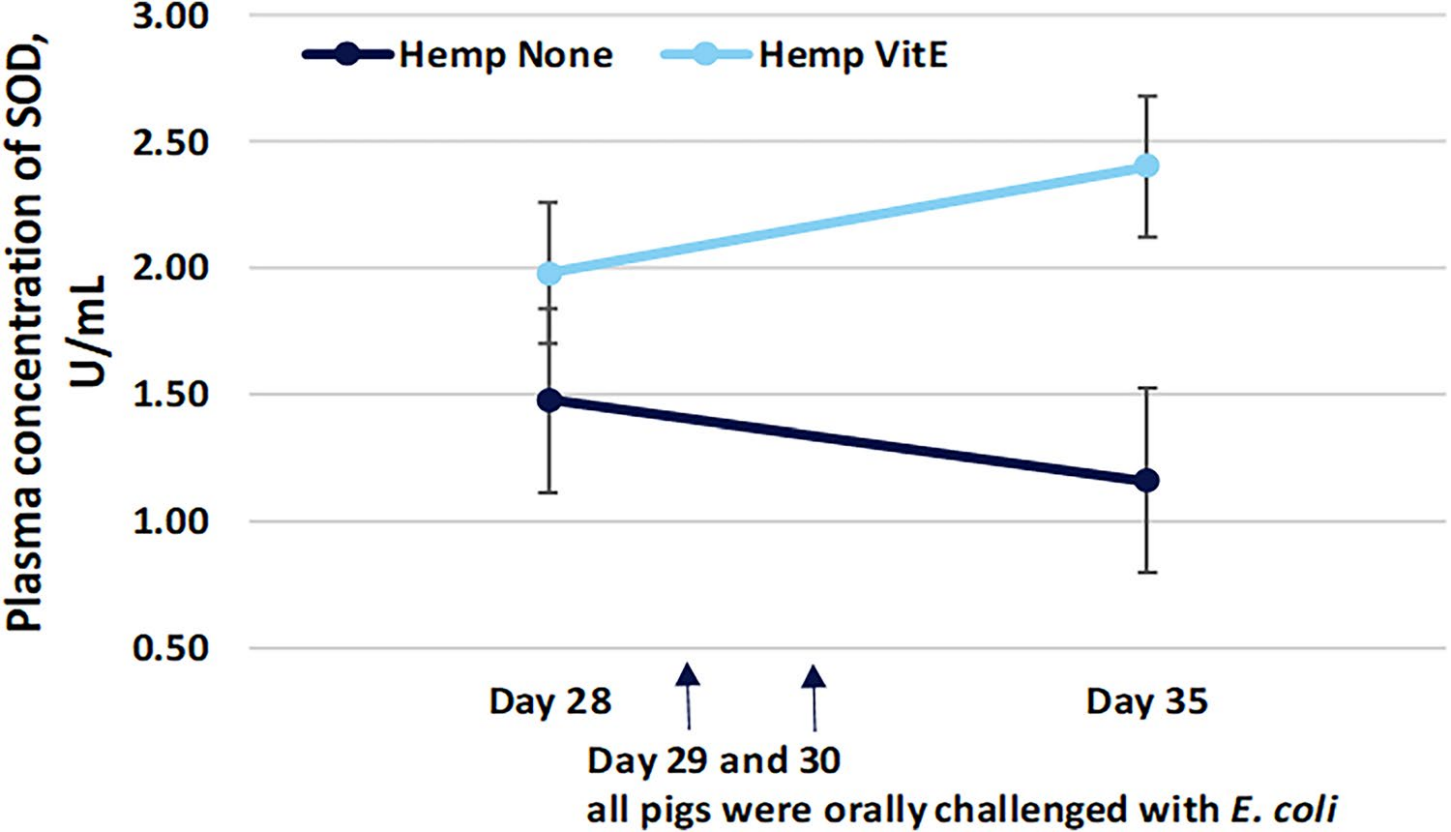
Interaction plot between day and supplementation of vitamin E showing that by combining hemp oil and vitamin E, the plasma concentration of SOD increased after *E. coli* challenge. Piglets were provided the oil treatments from day 10 of age until day 28 of age.

No effect of oil source nor vitamin E supplementation was observed in the plasma concentration of MDA before or after *E. coli* challenge (Table 6). At day 28 of age before *E. coli* challenge, the plasma concentration of SOD was lower (*P* = 0.03) in piglets provided vitamin E but only for piglets provided fish as oil source. In addition, a tendency (*P* = 0.08) of a greater SOD plasma concentration was observed in piglets allocated hemp oil supplemented with vitamin E compared to piglets allocated fish oil supplemented with vitamin E. At day 35 of age, the plasma concentration of SOD was greater in pigs provided hemp oil and vitamin E compared to pigs only provided hemp oil (*P* = 0.001). In addition, at day 35 of age, pigs provided hemp oil and vitamin E had a greater SOD plasma concentration than pigs provided fish oil and vitamin E (*P* = 0.001). An interaction between day and vitamin E supplementation was observed in the plasma concentration of SOD for both oil sources (*P* ≤ 0.03; Figs. 4 and 5). Furthermore, at day 35 of age after *E. coli* challenge, the plasma concentration of GPX1 was reduced in all pigs (*P* = 0.01).

**Table 6:** Effect of oil source and vitamin E supplementation on oxidative status in piglets pre- and post-*E. coli* challenge.

Oxidative stress marker	Treatment				SEM	*P* value		
	Fish	Hemp	Fish + vitamin E	Hemp + vitamin E		Oil source	Vitamin E	Day
MDA, µM								
Day 28	71.0	80.6	79.6	89.3	6.80	0.34	0.34	0.73
Day 35	78.4	91.8	73.6	87.0	14.0	0.75	0.75	
SOD, U/mL								
Day 28	1.57	1.48	1.03	1.98	0.282	0.08	0.03	0.19
Day 35	1.31	1.16	1.35	2.40	0.121	< 0.01	< 0.01	
GPX1, ng/mL								
Day 28	20.3	18.4	20.3	18.5	4.54	0.94	0.97	0.01
Day 35	8.81	12.3	11.1	14.7	3.20	0.64	0.64	

Values are presented as estimated marginal means and pooled standard errors (SEM), n = 6 piglets at day 28, and n = 4 piglets at day 35 of age. Plasma samples were obtained from piglets at day 28 (before *E. coli* challenge) and at day 35 of age (5–6 days after *E. coli* challenge). Piglets were provided oil treatments from day 10 of age to day 28 of age.

## Discussion

The aim of the current study was to investigate the effect of oil sources with different proportions of n-6 and n-3 PUFAs and supplementation of an antioxidant in the form of natural vitamin E on oxidative stress and immune responses in *E. coli* challenged piglets. Some LCPUFAs including n-3 fatty acids may reduce inflammatory reactions as they have shown anti-inflammatory properties, and fish oil has frequently been studied in relation to pig nutrition because of its anti-inflammatory and immunological potential^29,30^. In a previous study, hemp seed oil provision to sows resulted in direct maternal supply with LCPUFA, especially ALA and SDA, and piglets were able to convert these fatty acids obtained via the sow milk to C20:5n-3 and C22:5n-3^31^. In order to study this approach further, the treatments of the present study were based on hemp seed oil, which besides its content of ALA and SDA also contains bioactive compounds with antioxidant properties^21^. This oil was compared with fish oil characterized by containing C20:5n-3, C22:5n-3, and C22:6n-3 which are not present in vegetable oils. Both hemp oil and fish oil are characterized by a high content of PUFAs, which increase the risk of lipid oxidation in cellular membranes. Therefore, the effect of vitamin E addition in combination with the oil treatments was selected for the antioxidant treatment due its protective effect against PUFA oxidation and because of its bioactivity regarding modulation of host immune function^32^.

As expected, the hemp oil contained both n-6 and n-3 fatty acids but did not contain C20:5n-3, C22:5n-3, and C22:6n-3 as present in the fish oil used in this study. Piglets provided fish oil had a larger proportion of these n-3 fatty acids than piglets fed hemp oil at day 28 of age. The reason why plasma from piglets provided hemp oil contained these fatty acids could probably be ascribed to the maternal transfer while suckling^33^, and/or due to the metabolism of ALA and SDA by the piglets provided the hemp oil. The impact of the oil treatments on plasma fatty acid profile somewhat persisted until on day 35 of age. However, the influence of the oil treatments during the suckling was dimmed during the first week post-weaning when compared to day 28 of age, as also observed before^9^. It should be noted that the proportion of unsaturated fatty acids in plasma was higher after weaning than at weaning. Interestingly, piglets’ plasma rather than intestinal fatty acids and immunity was affected by the lipid treatments. This result was a surprise because in former studies^9,34^, a strong influence of dietary lipid nutrition of sows and piglets was observed on piglet responses pre- and post-weaning both with regard to incorporation of fatty acids and concentration of α-tocopherol in intestinal tissue and mucosa. It cannot be excluded that the oil supplementation provided orally to the piglet and not via the sow milk and feed is the reason for the outcome of this study, i.e., that the absorption and incorporation of fatty acids into intestinal tissue is more effective when added via the diet than via direct supplementation^35^.

After provision of vitamin E and before weaning/*E. coli* challenge, an improved antioxidative status was expected in the piglets^34^. The difference in vitamin E concentration of the oils was reflected in the plasma α-tocopherol concentrations during the suckling period except at weaning (day 28 of age). Especially for piglets at day 14 and 21 of age, provision of fish oil with vitamin E enhanced the α-tocopherol concentration in plasma, while for piglets provided hemp oil, provision of vitamin E enhanced the plasma α-tocopherol concentration towards the end of the suckling period. The obtained impact of vitamin E for the α-tocopherol concentration in plasma was expected from previous research on supplementary vitamin E for piglets^9^. Furthermore, the reduction in plasma α-tocopherol concentration after weaning and due to exposure to *E. coli* has also been observed previously^35^, and may indicate the biological activity of α-tocopherol during challenge of the immune system. It should be noted that the fish oil had a high amount of vitamin E compared to the hemp oil, but this was probably because the fish oil was a product designed for human supplementation, and that the product was enriched with a large amount of vitamin E to prevent oxidation. Although no difference in plasma α-tocopherol concentration was observed at day 28 of age between treatments, we obtained an increase in the INF-γ concentration of the plasma in piglets receiving vitamin E supplemented oils. This may be explained by the fact that immune cells of the blood may have had an increased α-tocopherol concentration, which upon stimulation in vitro with LPS influence the INF-γ production. Interleukin 12 was somewhat higher in plasma of piglets receiving hemp oil compared to piglets provided fish oil, while IL-18 tended to be higher in piglets receiving vitamin E supplemented oils. These interleukins can stimulate the production of INF-γ. Influence of dietary vitamin E and fatty acids on immunoglobulin concentration of piglets has previously been observed^9^. However, it is difficult to explain the increased IgG concentration in the pigs receiving vitamin E supplementation when provided fish oil at day 28 of age, whereas no effect was obtained for the hemp oil.

After weaning and *E. coli* challenge (on day 35 of age), the plasma concentration of the antioxidative enzyme GPX1 was reduced irrespective of the oil treatments as expected due to induction of oxidative stress as a result of the *E. coli* challenge and probably also due to the weaning process in general, involving changes in diet and environment^5^. The antioxidant enzyme GPX1 is highly expressed in the intestine and is involved in preventing accumulation of harmful intracellular hydrogen peroxide^36^. However, no impact of the oil treatment or age was obtained with regard to plasma MDA, which is a frequently used marker of oxidative stress even though the proportion of unsaturated fatty acids increased, and the concentration of α-tocopherol decreased in the plasma at day 35 of age compared to day 28 of age. Other studies^37,38^ observed slightly reduced MDA concentrations in blood plasma of pigs when providing treatments containing compounds with antioxidative properties. Furthermore, Jiang et al.^37^ and Zhao et al.^38^ did not observe an effect of antioxidant compounds (gallic acid and essential oils, respectively) on the antioxidative enzymes SOD and GSH-PX in plasma of pigs after induction of oxidative stress.

In the present study, the SOD plasma concentration was lower at day 28 of age in piglets provided vitamin E which was unexpected. However, for both oil treatments, an interaction between day and vitamin E supplementation was observed, i.e., after challenge with *E. coli*, the plasma concentration of SOD increased in piglets provided vitamin E, whereas the opposite was observed for piglets not provided vitamin E. This indicates that by combining oil source and vitamin E, the plasma concentration of SOD increases after challenge with *E. coli*, suggesting a better antioxidative status in piglets provided vitamin E in combination with the oil treatment. Furthermore, the plasma concentration of SOD was in general greater in piglets provided hemp oil compared to piglets provided fish oil, suggesting a better antioxidative response in piglets provided hemp as oil source.

In this study, we only examined plasma for oxidative stress responses. Plasma reflects the systemic concentration of antioxidative enzymes while mucus in the intestine reflects the local concentration of antioxidative enzymes. Since antioxidative enzymes unfortunately were not analyzed in intestinal tissue, it was not possible to investigate the local concentration of the antioxidant enzymes. Moreover, in the present study, all piglets were exposed to a mild infection with a mix of *E. coli* strains to reflect practical conditions. The mild infection of the piglets was reflected by the fecal results which showed mild diarrhea and limited *E. coli* shedding indicating that all piglets remained relatively healthy. The intention of this study was to examine the effect of vitamin E and fatty acids on oxidative stress and immune responses in piglets during practical conditions, i.e., all piglets were exposed to a mild infection of *E. coli*. In general, no effect was observed of oil source nor vitamin E supplementation on fecal shedding of *E. coli* or diarrhea incidence. However, fish oil seemed to lower the *E. coli* shedding compared to hemp oil, and vitamin E supplementation seemed to lower the fecal score. Overall, provision of oil treatment (in particular hemp oil rather than fish oil) in combination with supplemental vitamin E to suckling piglets may improve their oxidative status and immune responses and thus might reduce the risk of infection post-weaning and thereby the incidence of *E. coli* associated diarrhea.

## Conclusion

Oral provision of fish oil to suckling piglets enhanced the concentration of LCPUFAs in plasma rather than hemp oil, and supplementation of vitamin E to the oils enhanced the plasma α-tocopherol concentration. One week after weaning and exposure to *E. coli*, a general reduction in the α-tocopherol concentration and activity of GPX1 was obtained, while the proportion of the LCPUFAs were higher, and the overall proportion of unsaturated to saturated fatty acids in plasma were in general higher than at weaning. The oil treatments including vitamin E influenced immune responses at weaning, and provision of hemp oil (rather than fish oil) in combination with vitamin E enhanced the oxidative status post-weaning. However, more research is required to determine the impact of oxidative stress status in relation to immunity in pigs during an inflammatory response such as *E. coli* infection.

## Data Availability

The data that support the study findings are available upon request per mail to the corresponding author Pernille Aagaard Madsen, Email: pernille.madsen@anivet.au.dk.

## Abbreviations

ALA: α-Linolenic acid
DHA: Docosahexaenoic acid
EPA: Eicosapentaenoic acid
GLA: γ-Linolenic acid
GM-CSF: Granulocyte-macrophage colony-stimulating factor
GPX1: Glutathione peroxidase-1
IgA: Immunoglobulin A
IgG: Immunoglobulin G
IgM: Immunoglobulin M
INF-γ: Interferon-γ
IL: Interleukins
LCPUFA: Longer chained polyunsaturated fatty acids
MDA: Malondialdehyde
PUFAs: Polyunsaturated fatty acids
ROS: Reactive oxygen species
SDA: Stearidonic acid
SOD: Superoxide dismutase
TNF-α: Tumor necrosis factor-α
